# Mobile App–Based Smoking Cessation in Hispanic or Latino Adults: Culturally Tailored Spanish-Language Formative App Development Study

**DOI:** 10.2196/84249

**Published:** 2026-05-19

**Authors:** Ursula Martinez, Christine Vinci, Paula Hernandez, Marilyn Horta, Rebecca Blackwell, Thomas H Brandon, Vani N Simmons

**Affiliations:** 1 Department of Family and Preventive Medicine, University of Utah, 310 Wakara Way, Suite 1100, Salt Lake City, UT, 84108, United States, 1 801-213-2103; 2 Cancer Control and Population Sciences, Huntsman Cancer Institute, Salt Lake City, UT, United States; 3 Department of Health Outcomes and Behavior, Moffitt Cancer Center, Tampa, FL, United States; 4 Department of Psychology, University of South Florida, Tampa, FL, United States; 5 Department of Oncological Sciences, University of South Florida, Tampa, FL, United States; 6 Participant Research, Interventions, and Measurements (PRISM) Core, Moffitt Cancer Center, Tampa, FL, United States

**Keywords:** smoking cessation, mobile applications, digital health, mobile health, Hispanic or Latino, mobile phone

## Abstract

**Background:**

Despite the notable proliferation of smoking cessation mobile apps, to date, no validated, Spanish-language, culturally tailored mobile intervention exists for Spanish speakers in the United States.

**Objective:**

The aim of this study was to conduct formative research to inform the adaptation of an evidence-based smoking cessation intervention developed for Spanish-speaking Hispanic and Latino individuals from a printed format into a mobile app.

**Methods:**

Guided by a user-centered approach and in collaboration with product design industry experts, wireframes were developed to present the app’s layout and functionality. Focus groups were conducted over Zoom (Zoom Communications) with Spanish-speaking individuals who currently smoke to assess their previous mobile app experience, attitudes toward mobile apps, and feedback on app architecture and design. Two independent reviewers (RB in collaboration with another member from the qualitative core) trained in qualitative methods coded the focus group data using a thematic analysis approach and identified emerging themes.

**Results:**

The app wireframes included 4 navigation buttons on the home screen to organize and deliver evidence-based intervention content—Home (*Inicio*), Learn (*Aprende*), My Coach (*Mi Couch*), and Profile (*Perfil*). Different wireframe designs were generated in distinct color palettes. Data saturation was reached after three focus groups. Participants were 54% (7/13) women, had a mean age of 56 (SD 14.9) years, 39% (5/13) had an education ≤high school, and 31% (4/13) were married or cohabitating. All participants smoked daily, a mean of 14 (SD 7.8) cigarettes per day, for 32 (SD 16.9) years, and 54% (7/13) smoked ≤30 minutes of waking. Participants reported using social media, news, shopping, and gaming apps, but few used mobile health apps. Salient barriers for app use included worries regarding privacy breaches and fears about misinformation. Desired features included community-building elements, personalization, reward badges, knowledge checks, and audiovisual presentation of content within the app. Participants disliked having a countdown to quit date, preferring an “I quit” button to initiate monitoring progress. They also viewed sharing progress with support networks as a source of unwanted pressure, although a few saw it as motivational. Overall, participants liked the app design and indicated willingness to use it.

**Conclusions:**

This formative research provides critical insights into preferences related to the development of culturally tailored mobile smoking cessation interventions for Spanish-speaking individuals. Key findings highlighted enthusiasm for a smoking cessation app and the importance of including features that foster social connection and allow for personalization.

## Introduction

Hispanic and Latino individuals are the fastest-growing ethnic group in the United States, representing 19% of the population and projected to reach 28% by 2060 [1-3]. Although the overall prevalence of smoking among Hispanic and Latino individuals is lower compared with non-Hispanic White individuals [4], 4 out of the 5 leading causes of death in this population are related to smoking (ie, cancer, heart disease, cerebrovascular disease, and diabetes) [5]. Smoking rates also vary widely by Hispanic and Latino subgroups. For example, rates of smoking are particularly high among individuals from Cuban (31.3% men and 21.9% women) and Puerto Rican (35% men and 32.6% women) backgrounds [6], not only surpassing other Hispanic and Latino subgroups but even non-Hispanic Whites (12.9%) [4]. Additionally, Hispanic and Latino individuals encounter unique barriers to quitting smoking and have less access to and usage of evidence-based smoking cessation treatments [7]. Language is a significant challenge, as nearly one-third of Hispanic and Latino individuals speak English less than “very well” [8], and few smoking cessation resources are available in Spanish [9,10]. Thus, there is a great need to develop and validate culturally appropriate Spanish-language smoking cessation interventions.

Our evidence-based printed intervention was developed through a rigorous, multiphase transcreation process [11] involving formative focus groups to identify culturally relevant themes, translation and cultural adaptation of the Forever Free self-help materials [12], and iterative learner verification interviews assessing suitability, comprehension, cultural acceptability, and persuasion [13]. This intensive formative process resulted in the development of a self-help intervention for Hispanic and Latino individuals who preferred to receive their health information in Spanish [11]. It comprised 11 booklets and 9 pamphlets called *Libre del cigarrillo, por mi familia y por mí: Guía para dejar de fumar* (*Free from smoking, for my family and for me: Guide to quitting smoking*). Following principles of cognitive-behavioral therapy [14,15], these printed materials cover essential aspects of the smoking cessation process (eg, setting a quit date and behavioral and cognitive coping strategies) and were designed to be delivered on a monthly basis over the course of 18 months. A subsequent, fully powered, randomized controlled trial that involved 1417 Spanish-speaking Hispanic or Latino individuals nationwide demonstrated the efficacy of the Libre del cigarrillo intervention compared with an active control condition comprising the National Cancer Institute booklet, “*Guia: Viva de forma más saludable para usted y su familia, deje de fumar hoy mismo*” (*Live healthier for you and your family, quit smoking today*) [16,17], in increasing smoking abstinence across assessments every 6 months over the course of 24 months (eg, 33.1% abstinence for Libre del cigarrillo vs 24.3% for National Cancer Institute booklet at 24 months; odds ratio=1.54, 95% CI 1.18-2.02; *P*=.002) [18].

Despite the success of our intervention, printed materials have several limitations, such as their static content, reduced potential for engagement, and limited reach. The use of digital technologies has become increasingly popular in recent years to overcome these barriers, particularly mobile apps [19]. Advantages of using mobile apps include their ability to send time-sensitive intervention content in real time and in digestible portions. Users can also access content at any time from nearly anywhere. Apps can also send reminders and notifications that can be tailored to users’ preferences, and they can increase engagement and motivation through gamification [20]. Mobile apps are versatile, as they allow for changes when new content or updated information is available [21,22]. Smartphones are also widely available, with over 90% of the Hispanic and Latino US population owning one [23]. Thus, there has been an increase in the use of mobile apps designed to promote behavior change, and specifically, smoking cessation [24,25]. However, very few focused on the Hispanic and Latino population.

A review of the literature identified 23 studies that tested mobile health (mHealth) technologies among Hispanic and Latino individuals. Of those, only 8 used a smartphone app, and none focused on smoking cessation [26]. More recently, 2 studies have demonstrated the potential of mHealth smoking cessation interventions for Hispanic and Latino individuals. Cartujano-Barrera et al [27] found higher self-reported 7-day point-prevalence smoking abstinence among Latino adults who received a culturally accommodated SMS text messaging intervention in English or Spanish compared with those receiving standard-of-care educational materials. In addition, secondary analyses of an Acceptance and Commitment Therapy smoking cessation intervention delivered via a web platform [28] and a smartphone app [29] demonstrated efficacy among a subgroup of participants who identified as Hispanic or Latino. Notably, however, these latter interventions were available only in English.

Overall, mHealth smoking cessation interventions seem to be promising for Hispanic and Latino individuals. However, research is still scarce in this population, and in the United States, no evidence-based smoking cessation apps are available in Spanish. To address this gap, a multistep process was initiated to move the comprehensive written content of our previously validated, print-based smoking cessation self-help intervention and adapt it into an interactive mobile app. The intended users of the app are Hispanic or Latino individuals who prefer to receive their health information in Spanish. This study describes the foundational steps in the app development process, which included the creation of a comprehensive mobile app wireframe (ie, a blueprint of app layout and functionality) that includes both user experience and design elements (aim 1) and use of focus groups to gain in-depth insights into attitudes toward mobile apps, and to gather feedback to address the unique needs and preferences of Spanish-speaking Hispanic and Latino individuals who smoke in the United States (aim 2).

## Methods

### Participants

Participants were individuals who identified as Hispanic or Latino and who (1) reported smoking ≥5 tobacco cigarettes per week over the past year; (2) were ≥18 years old; (3) not currently enrolled in a face-to-face or virtual smoking cessation program; (4) were monolingual Spanish-speaking, or bilingual, Spanish-English, with a preference for receiving educational health materials in Spanish; (5) owned a smartphone; and (6) did not have another household member enrolled in the study.

### Procedures

Similar to the development of smoking cessation apps with other vulnerable populations [30,31], a user-centered design approach was followed to ensure the needs and preferences of the target population were addressed. First, we conducted formative research to understand the populations’ needs and preferences. Second, we used design principles relevant for the Hispanic and Latino population, as well as smoking cessation content from our previous efficacious self-help intervention [11,18]. Finally, we developed an app prototype in collaboration with industry experts in user design, who were bilingual and experienced with the target audience. This included selecting basic colors, fonts, and button styles; developing wireframes that display the functionality and flow of information; and determining preferences for user interactions with the app (aim 1).

The resulting wireframes were presented to participants in focus groups to receive their feedback and suggestions (aim 2). Participants were recruited via Craigslist and Facebook (Meta) advertisements. Individuals interested in the study were screened and consented over the phone by a study team member. After completing verbal informed consent, participants received a questionnaire online or by mail based on their preferences. The questionnaire had an estimated completion time of 15‐20 minutes. Upon receipt of the completed questionnaire, participants were invited to participate in a 90-minute focus group conducted via Zoom (Zoom Communications). Focus group sessions were conducted by female bilingual and bicultural members of the research team with graduate-level training in behavioral sciences or public health and with experience in qualitative data collection (RB and UM). At the time of the study, UM was an applied research scientist, and RB was a member of the Moffitt’s Participant Research, Interventions, and Measurement (PRISM) core. A project manager assisted with logistics and technology as needed and took detailed field notes during the sessions. No one else was present during the focus group sessions aside from participants and the research team. Participants were required to turn on their cameras and be in a private setting during the sessions. To support participation among individuals with varying levels of technology literacy, study staff provided written instructions in Spanish before each session and offered individualized technical assistance by phone as needed. No previous relationship was established between participants and researchers, and participants were only provided information about the study goals; the facilitators’ nationality and professional credentials were shared to establish trust and rapport. During focus group sessions, participants were encouraged to provide feedback and suggestions regarding potential features for the proposed mobile app. Data collected via focus groups were recorded and transcribed verbatim. All the transcripts were then translated to English by professional translation services. To preserve meaning and minimize loss of nuance, the certified English translations were reviewed by bilingual research staff, with professional training in linguistics and translation, and familiar with Hispanic and Latino cultural contexts and values. Discrepancies, ambiguities, or culturally specific expressions were discussed and resolved through consensus among bilingual research staff. The English transcripts were then analyzed to inform the development of a prototype app for testing. When needed, coders returned to the original Spanish transcripts for spot-checking during analysis to clarify meaning and ensure fidelity to participants’ intended perspectives [32].

### Measures

All measures were administered in Spanish and included the following:

- First, demographic information: demographic questions included items such as sex, gender, age, education, household size, debt level [33], country of origin, country of birth, and years living in the United States.
- Second, smoking history and nicotine dependence: included questions related to participants’ history of smoking, such as the number of cigarettes smoked per day and years spent smoking. Nicotine dependence was assessed using the Spanish-validated Fagerström Test for Nicotine Dependence [34].
- Third, readiness to quit smoking: to assess readiness to quit smoking, the 11-point Contemplation Ladder was used [35].
- Fourth, acculturation: to evaluate participants’ level of acculturation to the United States, the Short Acculturation Scale for Hispanics [36] was used. This measure is composed of 12 items and has shown high reliability across subgroups of Hispanic individuals [6].
- Fifth, smartphone usage: the smartphone usage subscale from the Media Technology Usage and Attitudes Scale [37] was used to evaluate the degree of smartphone use. This subscale is composed of 9 items evaluating smartphone use on a 10-point frequency scale from “never” to “all the time.”
- Finally, interview guide: a semistructured interview guide was developed to assess participants’ previous app experience (“what are examples of apps that you have on your smartphone?”), barriers (“what turns you off from using an app?"), and facilitators (“what motivates you to download an app?”) for app use, impressions and feedback on overall app design (“what do you think about the ‘Learn’ tab?”), and suggestions on app features (“what are your thoughts on receiving notifications on your phone?”). A copy of our interview guide has been included in Multimedia Appendix 1.

### Analyses

Data were analyzed using thematic analysis informed by principles of qualitative content analysis [38,39]. The analytic approach was primarily inductive, allowing themes to emerge from the data rather than being imposed a priori, while remaining informed by the study aims and previous literature on smoking cessation and mHealth interventions. Coding focused mainly on semantic (manifest) content by capturing participants’ explicit statements, while also attending to latent meanings that emerged through group interaction and contextual interpretation [40]. Participants’ responses and insights during the focus groups were analyzed by 2 expert coders (RB in collaboration with another qualitative core member) with relevant experience in thematic coding and qualitative analysis. The unit of analysis was meaningful excerpts from all focus groups, which were coded line by line by 2 independent coders (RB in collaboration with another qualitative core member) to draft an initial codebook. To assess intercoder reliability, 3 compiled documents were created, each consisting of aggregated meaningful excerpts drawn from all focus groups and representing every question and domain in the interview guide. These compiled excerpt documents were independently coded line by line by both coders as part of the reliability verification process, achieving substantial agreement (Cohen κ=0.81). Coding discrepancies were resolved through discussion until consensus was reached. The finalized codebook was then applied to all transcripts using NVivo 12 (Lumivero).

After coding, individual codes were organized into broader, higher-level categories according to conceptual relatedness. Themes were developed through an iterative analytic process involving team discussions, during which coded segments were reviewed and compared across focus groups. Themes were further refined to ensure conceptual clarity, internal consistency, and recurrence across groups and were chosen to represent patterns evident throughout the dataset rather than singular or atypical remarks [41]. Participants were not involved in the data analysis process.

### Ethical Considerations

The study was performed in line with the principles of the Declaration of Helsinki. Approval was granted by the Advarra Institutional Review Board (Pro00069694). Verbal informed consent was obtained from all individual participants included in the study. No identifying information is presented in this publication. All participants received a US $25 gift card after completing the questionnaire and an additional US $25 gift card upon completion of the focus group.

## Results

### Development of App Wireframe

During the App wireframe development, the goal was to preserve the core content from the existing evidence-based, culturally relevant Libre del cigarrillo booklets [11] by presenting it in “digestible” amounts. Therefore, the team selected app features that would promote engagement with the app while avoiding burden. Guided by these principles, the proposed app wireframe outlined the app layout and functionality, and it organized the content of the original self-help booklets into four categories: *Home* (*Inicio*), *Learn* (*Aprende*), *My Coach* (*Mi Coach*), and *Profile* (*Perfil*). The *Learn* tab hosted most of the content of the booklets, organized in “courses” that are further subdivided into “mini-classes” presented in a combination of text and videos. The *Learn* section also included quizzes with questions related to the content presented in each course. The *My Coach* tab could be customized by users with preferred strategies they can use to deal with urges to smoke (eg, a friend’s phone number and a guided meditation). Under *Profile*, users could provide personal information (eg, name, pictures, and money spent on cigarettes per week) to customize the app for a more personalized experience. Finally, in the *Home* tab, users were invited to set a quit date that starts a countdown and provided feedback on achievements, such as the number of courses completed, the number of days smoke-free, and the money saved after quitting smoking.

**Figure 1.**
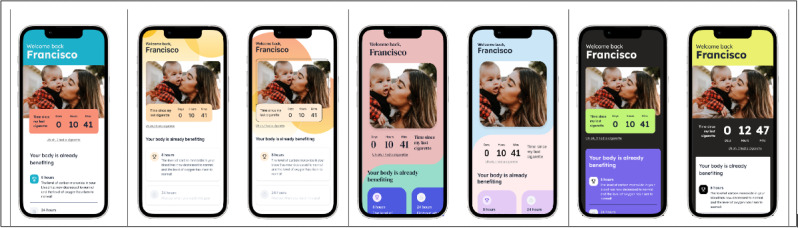
Examples of app wireframes showing design options.

To ensure cultural relevance, and consistent with our printed booklets, titles, and images across the app were designed to reflect core Hispanic and Latino values and characteristics. These included stories and photographs representing people from different backgrounds and nationalities, images of families (*familismo* or strong sense of attachment and loyalty to family) [42,43], and references to culturally relevant triggers, such as coffee consumption [11]. Given the importance of sense of community among Hispanic and Latino individuals [11], the app allows users to upload a personal photograph representing their reason for quitting (eg, family members) and to share app content and progress with friends or family to enhance social support. This feature will be entirely optional and fully controlled by the user. The team generated a variety of wireframe designs as well as in different color palettes. Figure 1 presents some examples of the final app wireframes.

### Focus Groups

A total of 23 individuals met the inclusion criteria, consented to participate in the study, and completed the baseline assessment. Of those, 10 did not attend the focus group sessions. Data saturation was reached after conducting 3 focus groups (n=13; 3‐5 participants per session). Participants’ mean age was 56 (SD 14.9) years, 54% (7/13) were women, 39% (5/13) had had an education of high school or lower, 31% (4/13) were married or cohabitating, and 39% (5/13) reported an annual household income lower than US $10,000. Most participants were born outside the United States or Puerto Rico, and most identified as Cuban, followed by Puerto Rican and South American. An average score of 18 on the Short Acculturation Scale for Hispanics indicated that acculturation to the United States was low (total score range 12‐60). Regarding smoking characteristics, all participants were daily smokers, and they had been smoking for an average of 32 (SD 16.9) years. They reported smoking about 14 (SD 7.8) cigarettes per day, and more than half of the sample smoked within 30 minutes of waking. Overall, participants were motivated to quit smoking, and they had an overall high level of smartphone usage. Table 1 provides more detailed information regarding sample characteristics.

**Table 1.** Sample characteristics (N=13).

Variables	Value
Sex (female), n (%)	7 (53.8)
Age (y), mean (SD)	56.0 (14.9)
Education (≤ high school), n (%)	5 (38.5)
Marital status (married/cohabitating), n (%)	4 (30.8)
Annual household income (<US $10,000), n (%)	5 (38.5)
Born outside of the United States or Puerto Rico, n (%)	11 (84.6)
Hispanic subethnicity, n (%)	
Cuban	7 (53.8)
Puerto Rican	2 (15.4)
South American	2 (15.4)
Central American	1 (7.7)
Spaniard	1 (7.7)
Race, n (%)	
White	8 (61.5)
More than one race	1 (7.7)
Do not know	3 (23.1)
Smoking characteristics	
Years smoking, mean (SD)	31.8 (16.9)
Frequency of smoking past 12 months (daily), n (%)	13 (100)
Cigarettes per day, mean (SD)	14.4 (7.8)
Smoke ≤30 minutes since waking, n (%)	7 (53.8)
Readiness to quit smoking (0‐10), mean (SD)	6.9 (2.8)
Short Acculturation Scale for Hispanics (12-60), mean (SD)	17.7 (8.3)
Smartphone usage (9-90), mean (SD)	54.8 (16.9)

### Themes

#### Overview

Findings from the focus groups were organized in five main themes: (1) prior app experience and perceived barriers, (2) app content, (3) app features: social support, (4) app features: notifications and app interactions, and (5) app design. Table 2 presents illustrative quotes within each theme.

**Table 2.** Themes and illustrative quotes from focus groups.

Theme	Sample interview content	Illustrative quotes
Prior app experience	What are examples of apps that you have on your smartphone? What do you like about using apps?	*“I have Facebook, WhatsApp, Instagram, and Twitter. All of them. But the ones I use the most are Facebook and WhatsApp.”* (Focus Group 1) *“I have WhatsApp because it makes communication with my family in Cuba much easier.”* (Focus Group 3) “*And the most important one that gets our money whenever they want, Supermarkert23*.” (Focus Group 3)
App content	Participants were asked to provide feedback on specific functions such as setting a quit date and mode of communication.	*“...in my case, this [setting a quit date] would cause me a lot of anxiety. I wouldn’t use this feature because it would increase my consumption of cigarettes, and the money I want to save by quitting I am going to spend right before. I am going to be in the red numbers in the app.”* (Focus Group 3) *“I would do it [setting a quit date], but I don’t know. I wouldn’t know when. For me, it would be good to count the days since you quit smoking. And from there, it [the app] can congratulate you.”* (Focus Group 2) *“Reading is a bit more tedious. So, on a video with audio, it keeps your mind busy, and you become more interested because you’re listening to someone tell you about a good experience.”* (Focus Group 2)
App features: social support	Are there ways you would like to get support from others?	*“I would share it. Almost all of my brothers smoke. But my family, my daughters, and friends are always telling me ‘quit smoking.’ So, I think that will motivate me even further because I will feel encouraged knowing that I am doing something to stop smoking. I wouldn’t mind.”* (Focus Group 1) *“I think I wouldn’t share it [quit date] with someone else unless they’re going through the same experience as I am. I would only share this with smokers.”* (Focus Group 3) *“I don’t think it would be a bad idea to maybe every week or two weeks, or once a month to have a chance to meet through the app. Because aside from learning, I’m having a great time with this group. I mean, I’ve laughed a lot. It’s a group that I feel bad about losing.”* (Focus Group 1) *“Maybe something like a circle that lets you connect with other people who also have the same problem with cigarette addiction and also want to quit.”* (Focus Group 2)
App features: notifications and app interactions	What are thoughts about receiving notifications on your phone? How would you like to track your smoking using the app?	*“If there’s an app that tells me every eight hours: “You’ve achieved this thing.” Then that is useful to me, and I like it.”* (Focus Group 1) *“As long as you don’t send me too many messages, it’s perfect. Two or three messages a day at the most. Because I get tired if they send me too many”* (Focus Group 1) *“You called once, twice, or even three times until we were all able to meet here today. But in the application, you’re given the information on what to do, but there’s no follow-up like, “How are you doing today? How much did you smoke today?” These things help patients or smokers more than information on a website.” (Focus Group 2*) *“There should be some form of follow-up. In some way, someone should question you or ask you to see your progress. To see whether you’re progressing.”* (Focus Group 2)
App design	We presented different app design options and invited participants to provide feedback to make the app more appealing	*“I liked [participant’s name] idea about uploading a picture that motivates you like an image of your grandson. If your motivation is to travel – but I think that each person can upload whatever they want to upload.”* (Focus Group 3) *“I mean, when you quit something it’s because you’re making a promise to someone or yourself, you know? It’s why they’re doing it. So, there could be an image of that. There could be a symbol of that so you can be reminded of that. “I made that promise, and that’s the reason why I am doing this.” It’s like willpower.”* (Focus Group 2) *“I don’t like color red. I don’t like anything red. So, if I see something red, I don’t even look at it.”* (Focus Group 2)

#### Prior App Experience and Perceived Barriers

Overall, participants viewed digital apps favorably. They highlighted the value of using mobile apps, especially social media platforms (eg, Facebook), messaging apps (eg, WhatsApp [Meta]), and remittance apps as valuable tools for community engagement, including the need to nurture transnational family ties. Some participants also described using news apps to stay informed, as well as gaming and shopping apps for leisure. However, some participants expressed feeling cautious or doubtful about content found on the internet. Identity theft and financial scams were also concerns. This distrust was more prevalent among participants who appeared to have moderate to low technology literacy. Notably, although participants did not report using mHealth apps, they expressed openness to adopting them in the future.

#### App Content

The “*Aprende*” or “*Learn*” feature garnered predominantly positive feedback across focus groups. Most found the feature beneficial for understanding smoking triggers and managing them. Participants particularly expressed enthusiasm to learn about smoking behaviors and the physiological effects of quitting. Across the focus groups, participants expressed a strong interest in understanding the health implications of smoking and the physical benefits associated with quitting. Many emphasized the value of communicating scientific knowledge, while also noting that not all information was equally relevant to all users. To enhance engagement, participants recommended including personalized features, such as videos or activities, to help distract users from the urge to smoke. Most participants had reservations regarding a “Quit Date Countdown,” fearing pressure and guilt if they could not quit by the set date. Participants expressed concerns that establishing a quit date in the app or sharing it with others could generate unhelpful anxiety and unwanted social pressure. Many suggested gradual, self-paced approaches, and preferred tracking the time between cigarettes or the decrease in the number of cigarettes smoked. Participants were more open to a “number of days smoke free” count *after* quitting smoking. Perceptions of the helpfulness of testimonials were mixed. Some participants referred to testimonies as inspiring, while others considered them unhelpful due to differences in personal experiences.

#### App Features: Social Support

A recurring theme throughout focus groups was participants’ desire to foster connections and build a sense of community. Participants frequently inquired about the potential inclusion of interpersonal and community-building features in the smoking cessation app. However, some displayed reservations about sharing their smoking cessation journey with family members, citing concerns about potential social pressure. Some participants, however, were open to sharing content in the app with family to help them understand emotional challenges during the postquit date period. Indeed, the *My Coach* feature – that generates notifications with individualized recommendations on how to manage difficulties during the cessation process – was seen as a potential “source of support,” with participants envisioning a virtual community where users facing cigarette addiction could connect and collectively work toward quitting. Whether through viewing success stories of individuals who quit smoking, sharing information with family members, or engaging in discussions with fellow app users, participants expressed a desire for human connection.

#### App Features: Notifications and App Interactions

There was a general enthusiasm for interactive features that could include educational material, motivational badges, progress tracking (eg, financial savings and reduction in the number of cigarettes smoked), and coaching advice, but there was also an emphasis on the need for personalizing the functions. In discussing *My Coach* and *Learn*, participants highlighted the importance of interaction within the app, whether through connecting with other app users, quizzes, or progress tracking, to keep their minds occupied and deter them from smoking due to boredom. Follow-ups to check if the user was able to resist the urge to smoke were also suggested to promote accountability. The feature of in-app badges received positive feedback across all focus groups, understood as motivating and engaging competitiveness without imposing control. Some participants suggested incorporating the impact of smoking on family members and caregivers through data tracking (eg, time spent smoking outdoors, which could otherwise be spent with grandchildren or nonsmoking relatives), providing a broader perspective on smoking consequences. SMS text messaging was considered more efficient in capturing attention for notifications. Finally, it was noted that excessive daily notifications deterred app usage, with 1‐2 daily notifications deemed optimal.

#### App Design

Given that our app design was based on our previous culturally relevant printed intervention [11,18] and included images that reflected diverse Hispanic and Latino subethnicities, food, and culture, no specific suggestions were provided on the app’s aesthetic features or appeal to Hispanic and Latino smokers specifically. Colors, images, and text style preferences varied. Some recommended an ability to personalize colors, and others said that images of nature could inspire everyone. One participant mentioned having negative reactions to the color red due to its association with oppressive political regimes in Latin America.

## Discussion

Despite the notable proliferation of smoking cessation apps, to date, no validated, culturally tailored app exists for Spanish speakers in the United States [44]. This study describes the results of formative research conducted to inform the adaptation of an extended evidence-based printed self-help intervention developed into a mobile app for Spanish-speaking Hispanic and Latino individuals.

In line with smoking cessation apps designed for the general population, the present wireframes included educational modules, development of a quit plan, ability to track progress, and rewards (eg, badges; Figure 1) [24,45]. Overall, participants thought that the wireframes were appealing. Most reported a wide variety of experiences using mobile apps. Therefore, the idea of receiving smoking cessation treatment in the form of an app was welcomed. Indeed, participants were excited about the possibility of receiving treatment content that could be personalized and interactive, and there was enthusiasm regarding progress tracking as well as receiving badges. These findings reflect the tremendous growth in internet use and access [46] as well as smartphone ownership among Hispanic and Latino individuals [23], which has opened the opportunity to use digital modalities to address health risk behaviors and potentially reduce health inequities [47].

Similar to the feedback received in our previous printed intervention development study [11], recommendations on how to improve the present app wireframes from participants in our study aligned with traditional values and social norms within the Hispanic and Latino population [48]. For example, participants suggested incorporating opportunities to build a sense of community and to establish personal connections with other fellow app users (*personalismo or the desire of building personal connections*) [43]. Although social support is important during smoking cessation treatment [10,49], we decided not to implement a feature that connects app users directly to protect privacy. However, users can share app content and their progress with their personal networks as desired. Another recurrent topic that emerged during our focus groups was related to family (*familismo or the importance of family*) [42,43]. Participants suggested including the beneficial impact of quitting smoking on the family. However, there were mixed feelings regarding sharing their smoking cessation journey (ie, data related to quitting) with family members to avoid social pressure. To allow for a sense of community and support while protecting privacy, each app user will have the choice to share specific educational content and progress with any family member or friend as they choose. This content will include different ways family and friends can support the users’ journey to becoming smoke-free (eg, social support).

An unanticipated result was related to a general hesitance toward having a countdown to the quit date within the app. Participants found this to be stressful and a source of discouragement if quitting was not achieved. It is worth noting that although motivation to quit smoking was overall high among participants in our sample, readiness to quit smoking was not required to participate in the study. Therefore, setting a quit date may have been perceived in this sample as premature and threatening, which may not be the case among individuals who choose to enroll in a smoking cessation program or download a smoking cessation app. In a recently developed culturally and linguistically adapted text messaging intervention for Latino individuals who smoke, Cartujano-Barrera et al [27,50] included a “Decision Support” component that provided guidance to develop a quit plan and select a quit date among participants who were not ready to quit smoking. This type of approach may be particularly beneficial to avoid feelings of pressure to quit. Therefore, in line with the Clinical Practice Guidelines for Tobacco Use and Dependence [10], we will recommend our app users to set a quit date, but we will also provide guidance and support through the process to minimize potential pressure and stress. Additionally, as suggested by study participants, the app will also incorporate a count-up feature in the *Profile* tab that can help interested users keep track of the number of days smoke-free.

Finally, to address participants’ privacy concerns, the app will be distributed via a secure link for app download rather than requiring a username and password, similar to another app developed by the research team [51]. This approach reduces access barriers and minimizes participant burden. In addition, secure links minimize credential exposure, thereby enhancing security. Secure links can incorporate strong cryptographic tokens such as short-lived, one-time tokens that offer greater protection against phishing and are safer than static passwords. We will also keep personal information to a minimum and optional based on participant preferences. For example, users may choose to use a pseudonym instead of their real name or may wish to upload photos to personalize the app. In addition, all app users will also have access to a detailed explanation of how their data will be stored, managed, and protected.

Findings from this study will inform next steps in this multiphase development process. The proposed app will condense the content of all 11 booklets and 9 pamphlets from our previous evidence-based printed self-help intervention into 7‐9 modules and will use various interactive media formats to enhance users’ experience (ie, text, audio, and videos). For example, we will include videos featuring personal testimonials from Hispanic or Latino individuals representing different subethnicities. Importantly, based on participants’ feedback and in line with Hispanic and Latino traditional values [48], the app will incorporate features that foster social connection and allow for personalization. Users will be able to personalize the app with their preferred coping strategies aligned with their cultural context and values. Given the limited availability and barriers to behavior change interventions using digital technologies for Hispanic and Latino individuals [26,52], lessons learned from our study can guide the development of innovative, culturally tailored treatments that address the specific needs of this vulnerable population.

Limitations of this study include the modest sample size, in which participants with Cuban background were overrepresented. It is noteworthy, however, that Cubans have high rates of smoking and experience more challenges quitting smoking compared with other Hispanic and Latino subethnicities [53]. Only individuals who preferred to receive educational materials in Spanish were included in the study, which may not represent Hispanic and Latino individuals who may be more acculturated and less proficient in the Spanish language. Furthermore, all focus group sessions were conducted via Zoom, which may have deterred individuals with low technology literacy from participating.

Culturally adapted interventions are typically more acceptable for populations that do not identify with general cultural norms, and there has been evidence suggesting that they can improve smoking cessation rates in these specific populations when compared with standard treatments [27]. However, there are limited evidence-based smoking cessation resources for Hispanic and Latino individuals [9], especially in the realm of digital interventions [44]. This study represents key foundational steps to develop a culturally relevant Spanish-language app to address smoking cessation among Hispanic and Latino individuals. Results indicate that Spanish-speaking Hispanic or Latino individuals in our sample were open to using a smartphone app. They also showed interest in psychoeducational content delivered via a variety of audio-visual formats, as well as personalizing and interacting with the app through badges and rewards. Finally, our results highlight the importance of incorporating features and content that align with core Hispanic and Latino values, such as those that foster social connection and allow for personalization. These findings can help inform future culturally relevant smartphone app development with this population.

## Supplementary material

10.2196/84249 Multimedia Appendix 1 Focus group interview guide.

10.2196/84249 Checklist 1 COREQ checklist.

## Abbreviations

mHealth: mobile health
PRISM: Participant Research, Interventions, and Measurement
